# Machine Learning Using Template-Based-Predicted Structure of Haemagglutinin Predicts Pathogenicity of Avian Influenza

**DOI:** 10.4014/jmb.2405.05022

**Published:** 2024-08-06

**Authors:** Jong Hyun Shin, Sun Ju Kim, Gwanghun Kim, Hang-Rae Kim, Kwan Soo Ko

**Affiliations:** 1 Department of Microbiology, Sungkyunkwan University School of Medicine, Suwon 16419, Republic of Korea; 2 Department of Biomedical Sciences; BK21 FOUR Biomedical Science Project; Medical Research Institute, Seoul National University College of Medicine, Seoul 03080, Republic of Korea; 3 Department of Biomedical Sciences; Department of Anatomy & Cell Biology; BK21 FOUR Biomedical Science Project; Medical Research Institute, Seoul National University College of Medicine, Seoul 03080, Republic of Korea

**Keywords:** Convolutional neural network, principal component analysis, abnormality detection, machine learning, avian influenza, haemagglutinin

## Abstract

Deep learning presents a promising approach to complex biological classifications, contingent upon the availability of well-curated datasets. This study addresses the challenge of analyzing three-dimensional protein structures by introducing a novel pipeline that utilizes open-source tools to convert protein structures into a format amenable to computational analysis. Applying a two-dimensional convolutional neural network (CNN) to a dataset of 12,143 avian influenza virus genomes from 64 countries, encompassing 119 hemagglutinin (HA) and neuraminidase (NA) types, we achieved significant classification accuracy. The pathogenicity was determined based on the presence of H5 or H7 subtypes, and our models, ranging from zero to six mid-layers, indicated that a four-layer model most effectively identified highly pathogenic strains, with accuracies over 0.9. To enhance our approach, we incorporated Principal Component Analysis (PCA) for dimensionality reduction and one-class SVM for abnormality detection, improving model robustness through bootstrapping. Furthermore, the K-nearest neighbor (K-NN) algorithm was fine-tuned via hyperparameter optimization to corroborate the findings. The PCA identified distinct clustering for pathogenic HA, yielding an AUC of up to 0.85. The optimized K-NN model demonstrated an impressive accuracy between 0.96 and 0.97. These combined methodologies underscore our deep learning framework's capacity for rapid and precise identification of pathogenic avian influenza strains, thus providing a critical tool for managing global avian influenza threats.

## Introduction

Influenza viruses belong to the Orthomyxoviridae family, and consist of segmented genomes of negative-sense single-stranded RNA [1]. There are four main species of influenza viruses, namely A, B, C, and D. The range of hosts is determined by the glycan moiety of the sialic acid receptors. Although each species has various host ranges, type A is known to have the most variable host range, including aquatic birds [2]. In particular, humans and pigs are the common hosts of all four types; thus, pigs are important mediators of influenza adaptation and transmission to humans [3].

Avian influenza is one of the most important threats to domestic poultry, and is also potentially zoonotic, making it capable of causing a pandemic [4]. For example, the 1918 pandemic caused by the H1N1 influenza virus, which is estimated to have infected one-third of the world population and killed at least 50 million people, have been recognized to have an avian origin [5]. Avian influenza type A viruses are classified into subtypes based on two surface proteins, hemagglutinin (HA) and neuraminidase (NA). There are 16 HA subtypes (H1 to H16) and 9 NA subtypes (N1 to N9), and diverse combinations of HA and NA proteins are possible. Avian influenza viruses are divided into “low pathogenic” or “highly pathogenic”, based on their genetic features and the severity of the disease causing poultry. Although several subtypes (*e.g.*, H5N1) are known to be highly pathogenic avian influenza (HPAI), the association between subtype and pathogenicity is not absolute. As HPAI severe disease and death in poultry and probably in humans, it is important to detect and response to HPAI quickly.

Machine learning and artificial intelligence have been applied on various data, and have demonstrated their ability to characterize complex biological data. In particular, the recent explosion of multi-omics data demands alternative approaches to overcome the challenge of “Data Deluge”, and to derive meaningful conclusions from the analysis of biological data [6]. Deep-learning methods are a type of representation learning methods, which obtain multiple levels of representation by combining multiple simple but non-linear modules, each of which transforms the representation at one level (starting with the raw input) into a representation at a higher, slightly more abstract level [7]. The application of machine learning for analyzing protein structure has been hindered by the lack of a transformation method, for transforming protein sequences into a computer-accessible format that preserves the structural information [8].

In this study, we not only transform protein sequences into a computational format using a two-dimensional CNN but also extend our analysis to include PCA and one-class SVM for refined abnormality detection and classification, enhancing the predictive power for pathogenicity in avian influenza viruses.

## Materials and Methods

### Retrieval of Avian Influenza Genome Sequences

The genome sequences of avian influenza viruses for training were retrieved from Avian Influenza DataBase (AIDB; http://avian-flu.org) on 27 December 2018. A total of 12143 whole genome sequences were deposited in the local server. The HA and NA (HN) types were also noted for further analyses.

### Translation of Avian Influenza Genomes

The genome sequences were translated and annotated using vigor4 (vigor-4.1.20190810-021720-7fa683e) [9]. Each segment 4 encoded two proteins, namely hemagglutinins HA1 and HA2.

### Template-Based Prediction of Haemagglutinin Structures

The structures of HA1 and HA2 were predicted using Modeller [10]. The template structure of 3HTO, the X-ray diffraction crystal structure of hemagglutinin of the avian H1N1 influenza A virus [11], was retrieved from the RCSB Protein Data Bank (PDB). The amino acid sequences were aligned using MAFFT (v7.475) [12], And the prediction models were automatically selected by Modeller. Superimposations of predicted structures against the template structure were depicted using PyMOL (PyMOL 2.4, Schrödinger).

### Structure of Models

The models consisted of an input layer with image transformation, a 2D convolutional neural network with 64 filters, a rectified linear unit, and a max pooling layer. Models 0 to 6 involved the addition of layers identical to the input layer according to the model number. The output layer consisted of flatten, dense, rectified linear unit, dropout, dense, and sigmoid layers. The results were indicated using 1-hot encoding, with 1 for pathogenic, and 0 for neutral. The maximum epoch was set to 200, with a batch size of 16. To prevent overfitting, early stopping was applied when validation loss backpropagated with a minimum delta of 0.01 more than 10 times [13].

### Selection and Post-Hoc Evaluation of Best-Fit Model for Supervised Learning

The best-fit model was selected from among the seven models according to the accuracy and loss of training results. Then, 30 sequences of avian influenza genome segment 4 that were reported as highly pathogenic were used for post-hoc evaluation. The result was indicated as a score, calculated as the difference in accuracy and misclassification multiplied by total samples [14].

### Principal Component Analysis (PCA)-Based Abnormality Detection and KNN Model Performance Evaluation

We analyzed the complete structural information of the predicted HA protein using Principal Component Analysis (PCA). Initially, we converted the three-dimensional structural information of the protein into numerical data using the coordinates of the C-alpha atom of the amino acids. Since the length of atoms varies with different proteins, we employed zero-padding to equalize their lengths. Subsequently, we standardized the data using the StandardScaler from sklearn library in Python. Then, we performed PCA, setting the number of principal components to four, using sklearn PCA library.

With the extracted principal components, we carried out an abnormality detection. We employed the one-class SVM model for the detection. To prevent overfitting and to achieve more robust results, we generated a Receiver Operating Characteristic (ROC) curve based on averages through 100 bootstrap replications. The standard deviation was represented as a shaded area in the ROC curve. Subsequently, we validated the distinction between neutral HA and pathogenic HA using K-nearest neighbor (K-NN) method. We utilized GridSearchCV for cross-validation of the K-NN algorithm and to prevent overfitting, identifying the optimal hyperparameters. Employing bootstrap analysis, we repeatedly assessed the model's performance and subsequently calculated the average and standard deviation of the performance metrics [15, 16].

## Results

### Characteristics of the Dataset

The dataset consisted of the whole genome sequences of 12,143 avian influenza samples. All isolates were sourced from birds, with Anatidae and Gallus gallus, consisting of more than 50% of the total samples. The samples were isolated from 64 countries, but the USA and China accounted for more than 50%, with 5,270 and 2,561 samples, respectively. A total of 119 HN types were found, including H9N2, H4N6, H3N8, H5N1, and H5N2, accounting for more than five hundred samples each.

Owing to the lack of meta-data, especially with regard to pathogenicity of the viruses, the viruses were classified into two classes, namely pathogenic and neutral, based on their HN types, with the virus being classified pathogenic when HN types were H5N1 and H7N7 because of known human outbreaks by the types [17, 18].

### Transformation of Nucleotide Sequences

Transformation of nucleotide sequences involved three steps. First, whole genome nucleotide sequences were translated into amino acid sequences, then the structures of HA1 and HA2 were predicted from that of the template 3HTO. Finally, the surfaces of the predicted structures were drawn. In each step, the average size of the dataset was 1.446, 0.034, 157.479, and 1,515.302 kb, respectively.

### Results of Machine Learning

The machine learning models consisted of zero to six mid-layers, along with input and output layers. The average of the final epochs was 18.429, and the median was 17. The averages of loss of training set, accuracy of training set, loss of validation set, and accuracy of validation set, were 0.240, 0.927, 0.422, and 0.928, respectively. Their medians were 0.243, 0.927, 0.294, and 0.956, respectively (Table 1).[Fig F1]

### Best-Fit Model for Supervised Learning

**Fig. 2:**
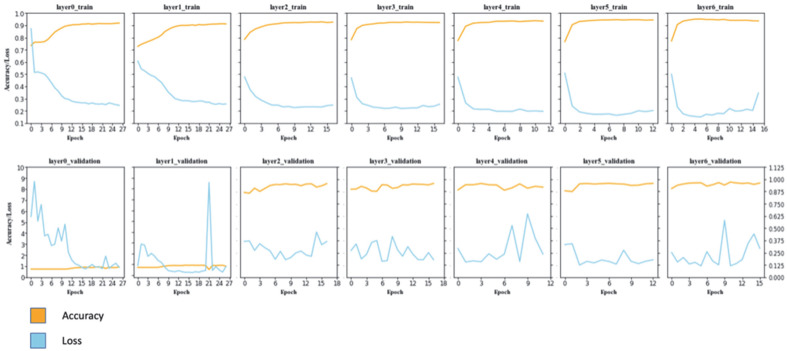
Learning curve of the models. The values are indicated by each epoch. Plots colored in orange show accuracy, in blue show loss per epoch. Accuracy shows the accuracy of model, matching ratio of the prediction and result, greater is better and loss shows absolute loss function, the absolute difference of the result and prediction, lesser is better.

For a better understanding of the progress of learning, learning curves for the training and validation sets were drawn (Fig. 2). The accuracy and loss of validation sets showed backpropagation and were early stopped after 30 epochs. Accuracies were over 0.9 in all except for the accuracy of the validation set of the model with one layer, which was 0.8357.

The model with six mid-layers showed the highest accuracy in both the training and validation sets, while the model with one mid-layer showed the lowest accuracy. The learning curves of the validation sets showed that the models with no mid-layer and one layer had a larger loss in validation sets than the other five models.

The models with 0, 1, and 4 mid-layers showed higher accuracy in the training sets than in the validation sets, indicating overfitting. Based on the learning curves and final accuracy with loss, the model with five layers was estimated to be the best-fit model.

### Evaluation of Best-Fit Model

As the classification of the datasets was ambiguous owing to the lack of meta-data, further evaluation was used to confirm the accuracy of the models. Thirty nucleotide sequences including non-H5N1 and non-H7N7 types, which were confirmed to be highly pathogenic avian influenza viruses by their metadata from NCBI, were used to evaluate the models. The sequences were transformed into a suitable image following the methods described above and provided as input data to each model. Thirteen out of 30 sequences were classified as those of highly pathogenic avian influenza viruses.

The output was 1-hot encoded as the classification matched the metadata (Table 2). The scores ranged from 12 to 24, with a median of 17. The model with one layer showed the lowest score, while the model with four layers showed the highest score. Notably, the model with four layers classified the highly pathogenic samples much more accurately than the other models.

### Principal Component Analysis-Based Abnormality Detection

**Fig. 3:**
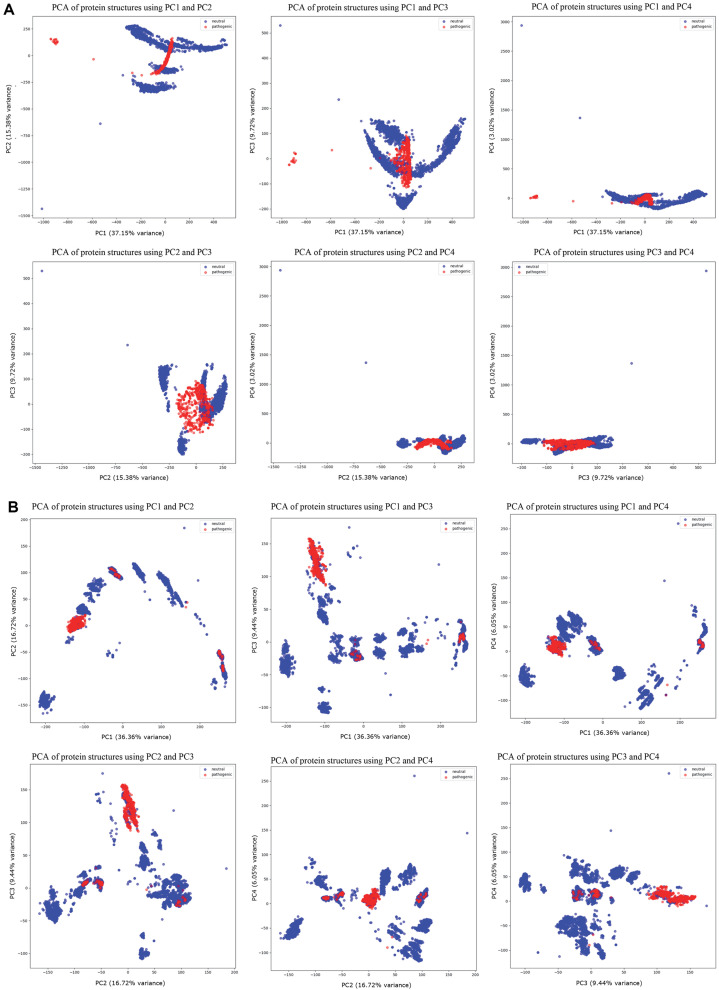
Plot based on principal component combinations of HA1 (A) and HA2 (B).

We observed distinct patterns across all PCAs. Pathogenic HAs were notably concentrated in specific regions. This pattern was consistently observed in both HA1 and HA2. In the PCAs for HA1, clusters of neutral and pathogenic HAs were present in the combination of PC4 with the other principal components, but they were not notably significant (Fig. 3A). In contrast, the PCAs for HA2 showed meaningful cluster differences in all PC combinations (Fig. 3B).

**Fig. 4:**
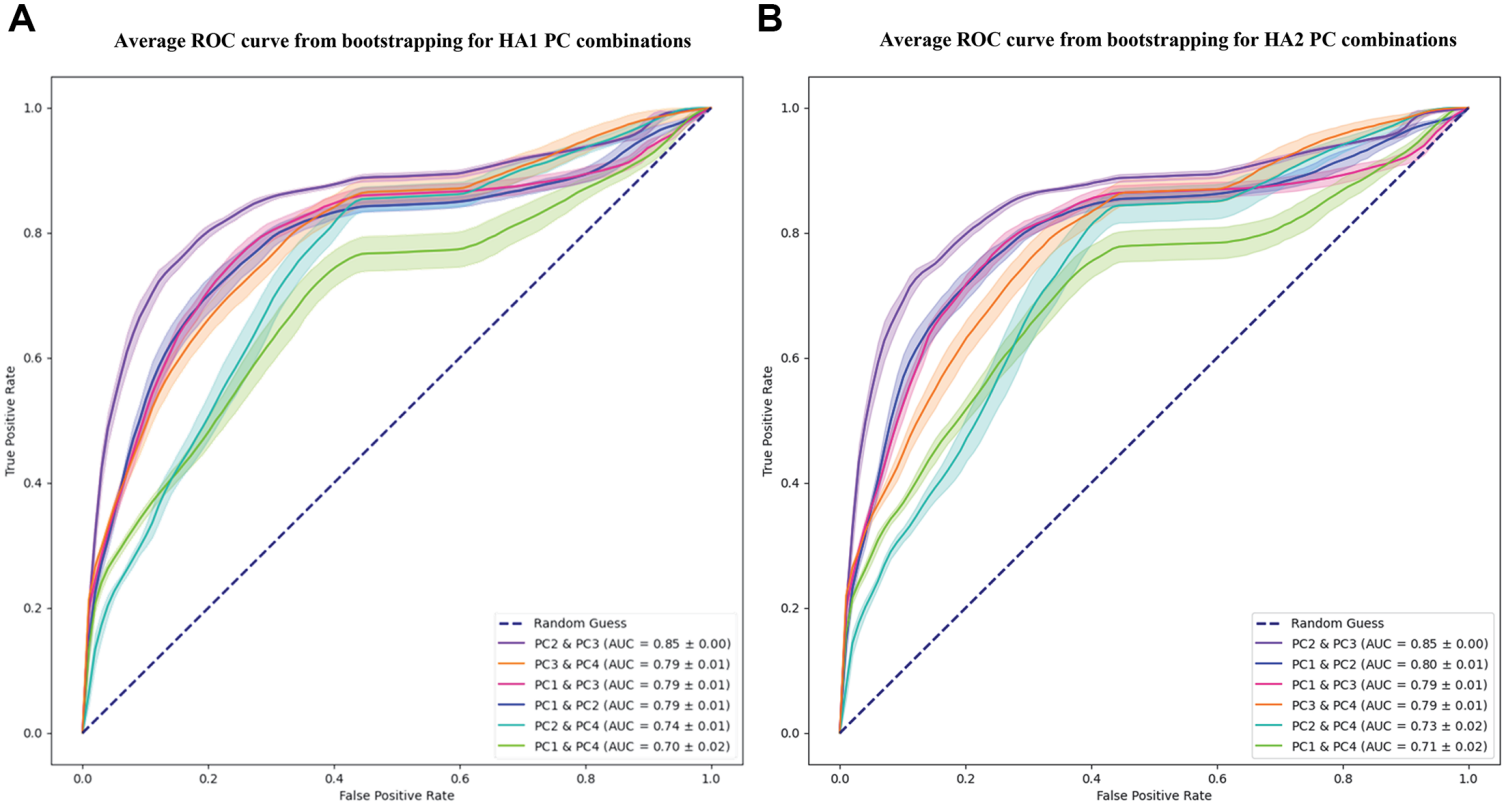
ROC curves for abnormality detection of HA1 (A) and HA2 (B) with 1,000 bootstrapping replications. Standard deviation was shaded on the lines for each principal component combination.

Based on the PCAs and the distinct structural clustering, we applied an abnormality detection method. The ROC curve for abnormality detection was represented by the average of 100 bootstrapping iterations, and the standard deviation was shaded on the lines for each principal component combination. In the ROC curve, both HA1 and HA2 exhibited that the combination of PC2 and PC3 excelled with an AUC value of 0.85 (Fig. 4). However, the combination of PC1 and PC4 displayed the lowest scores with AUC values of 0.70 and 0.71, respectively.

### Evaluation of the K-NN Model

K-NN model was applied based on the results of PCA. The number of neighbors considered ranged from odd numbers between 3 and 11. Equal weights were applied to all neighbors, and weights were assigned inversely proportional to the distance of the neighbors. GridSearchCV was employed with 5-fold cross-validation to prevent overfitting and to identify the optimal hyperparameters. 1,000 bootstrapping replications were performed. Consistent with the results of abnormality detection, the combination of PC2 and PC3 had the highest accuracy (Table 3). In contrast, the combination of PC3 and PC4, which had the lowest AUC value, ranked second in terms of accuracy.

## Discussion

The application of machine learning in biology, exemplified by projects such as AlphaFold, has been extraordinarily successful in diverse fields, from the development of new drug targets to predicting infestation outbreaks [19]. However, preparing an adequate dataset to build precise models requires considerable effort. A total of 51,343 viral genomes have been curated by NCBI, out of which only 29,810 are complete and annotated. What further makes applying machine learning in biology difficult is that the genome data are unlabelled, and thus have little or no information regarding characteristics that are of research interest, such as virulence, pathogenicity, and host range.

In addition to strictly curating databases and increasing the sharing of genome sequences, adopting semi-supervised learning is also worth considering [20]. Semi-supervised learning combines part of a labelled dataset with an unlabelled dataset to build a model. For example, training models with actual labelled data will increase their accuracy, revealing previously ignored characteristics that determine the pathogenicity of avian influenza viruses.

This study demonstrates the application of a convolutional neural network for supervised learning of protein structures, using set of avian influenza virus whole genome sequences. The resulting models with zero to six mid-layers had accuracies of over 0.9, except for the model with one mid-layer.

Further evaluation of models with 30 samples which were not H5N1 or H7N7 showed an accuracy ranging from 0.4 to 0.8. It is notable that the model trained on artificially labelled datasets can correctly classify the actual dataset, and application of other machine learning methods, such as reinforcing learning, could reveal uncharacterized alterations in HA protein, that could induce pathogenicity.

We also evaluated the performance of unsupervised learning to segregate groups into neutral and pathogenic based on the entire structure of the HA protein. Abnormality detection demonstrated the accuracy ranging between 0.7 and 0.85, and the K-NN approach showed the accuracy between 0.96 and 0.97. Despite the measures taken for overfitting prevention and cross-validation, the models yielded remarkably high accuracy even in the absence of labels such as pathogenic information.

Although our study has some limitations, including the lack of specific pathogenic information, the discovery of a model that can rapidly detect pathogenic avian influenza viruses through the structure of the HA protein, which is known to determine pathogenicity, would be significant. Further research and refinement of the model, along with the incorporation of more data, would be necessary to counter the global concern of pathogenic avian influenza.

## Supplemental Materials

Supplementary data for this paper are available on-line only at http://jmb.or.kr.



## Figures and Tables

**Fig. 1 F1:**
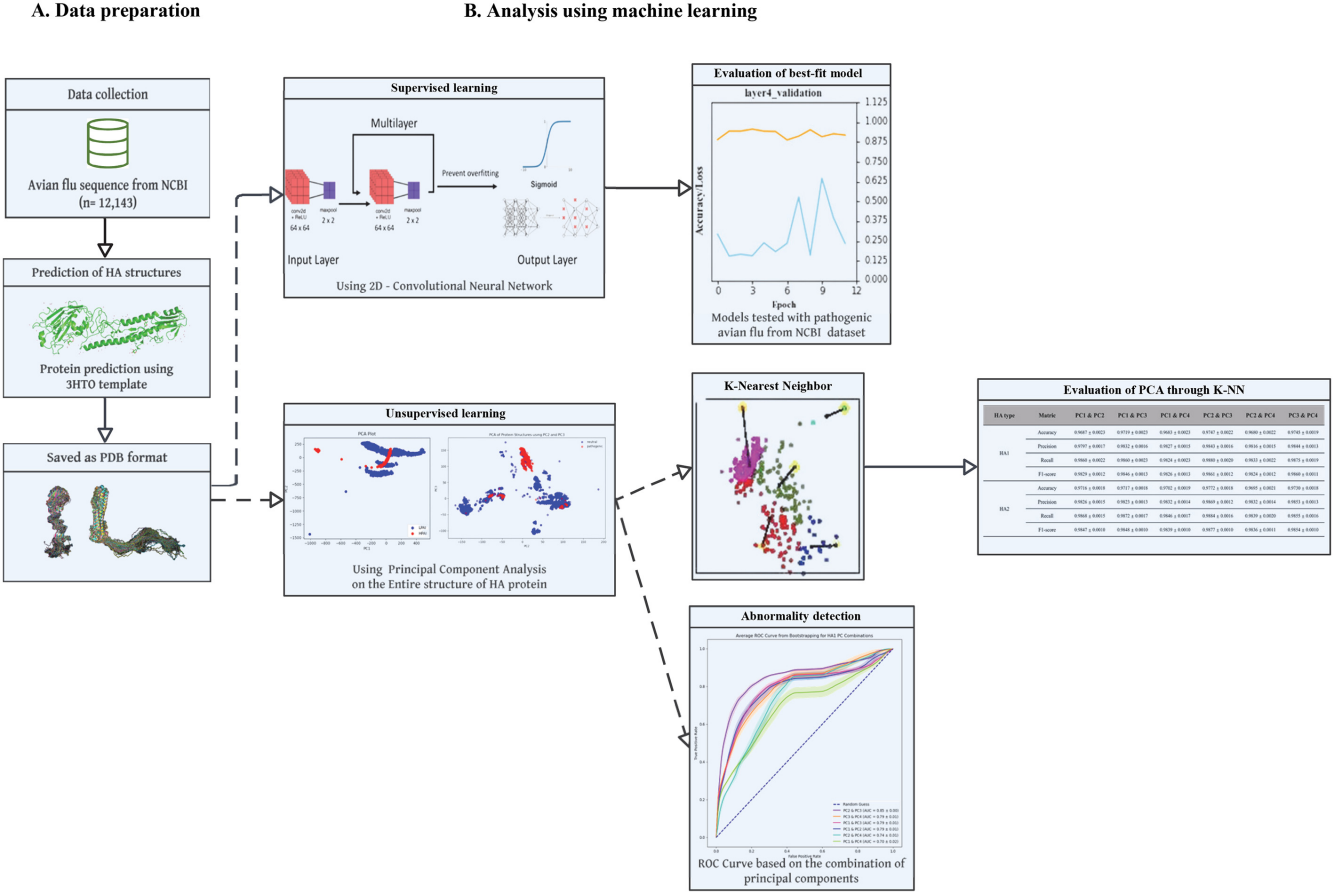
Flowchart of the entire process.

**Table 1:** Overview of the supervised learning models.

# of mid-layers	Final epoch	Training set		Validation set	
		Loss	Accuracy	Loss	Accuracy
0	27	0.2483	0.9169	0.8910	0.9159
1	27	0.2566	0.9081	0.8182	0.8357
2	17	0.2425	0.9270	0.3645	0.9556
3	17	0.2397	0.8251	0.1777	0.9573
4	12	0.1958	0.9339	0.2349	0.9104
5	13	0.2075	0.9401	0.1773	0.9577
6	16	0.2861	0.9349	0.2936	0.9610

**Table 2:** Post-hoc evaluation of the models with avian influenza viruses known to be highly pathogenic.

Accession No.	HN type	Virulence	Number of mid-layers ^a^						
			0	1	2	3	4	5	6
MW822903	H5N6	HPAI	1	1	1	1	1	0	1
MW822911	H5N6	HPAI	1	1	1	1	1	0	1
JQ041399	H5N2	HPAI	1	1	0	0	1	0	1
JQ041402	H5N2	HPAI	1	1	0	0	0	0	0
KP714480	H5N2	HPAI	0	1	0	0	1	0	0
GU727661	H5N5	HPAI	0	1	0	0	0	0	0
KP090439	H5N6	HPAI	0	1	0	1	1	0	0
KJ413834	H5N8	HPAI	0	1	0	0	1	0	0
KJ476669	H5N8	HPAI	0	1	0	0	1	0	0
KJ476670	H5N8	HPAI	0	1	0	0	1	0	0
JX397993	H7N3	HPAI	0	1	0	0	1	0	0
KU558906	H7N8	HPAI	0	1	0	0	0	0	0
MF357804	H7N9	HPAI	0	1	0	0	0	0	0
EPI1665336	H5N1	.	0	1	0	0	0	0	0
EPI1665344	H5N1	.	0	1	0	0	0	0	0
EPI1665360	H5N1	.	0	1	0	0	0	0	0
EPI1665368	H5N1	.	0	0	0	0	0	0	0
EPI1665320	H5N6	.	0	1	0	0	0	0	0
EPI1665328	H5N6	.	0	1	0	0	0	0	0
EPI1665392	H5N6	.	0	1	0	1	0	0	0
EPI1478981	H6N8	.	0	1	0	0	0	0	0
EPI1478989	H7N7	.	0	1	0	0	0	0	0
EPI1675506	H9N2	.	0	1	0	0	0	0	0
EPI1675564	H9N2	.	0	1	0	1	0	0	0
EPI1675572	H9N2	.	0	1	0	0	0	0	0
EPI1676342	H9N2	.	0	1	0	1	0	0	0
EPI1676350	H9N2	.	0	1	0	1	0	0	0
EPI1676358	H9N2	.	0	1	0	1	0	0	0
EPI1676398	H9N2	.	1	1	0	0	0	0	0
EPI1676406	H9N2	.	1	1	0	0	0	0	0

^a^The results are notated in one-hot encoding, 0 indicates the prediction not matches the result, while 1 indicates the prediction and the result matches.

**Table 3:** Evaluation of model performance using K-NN model with principal component combinations.

HA type	Matric	PC1 & PC2	PC1 & PC3	PC1 & PC4	PC2 & PC3	PC2 & PC4	PC3 & PC4
HA1	Accuracy	0.9687±0.0023	0.9719±0.0023	0.9683±0.0023	0.9747±0.0022	0.9680±0.0022	0.9745±0.0019
	Precision	0.9797±0.0017	0.9832±0.0016	0.9827±0.0015	0.9843±0.0016	0.9816±0.0015	0.9844±0.0013
	Recall	0.9860±0.0022	0.9860±0.0023	0.9824±0.0023	0.9880±0.0020	0.9833±0.0022	0.9875±0.0019
	F1-score	0.9829±0.0012	0.9846±0.0013	0.9826±0.0013	0.9861±0.0012	0.9824±0.0012	0.9860±0.0011
HA2	Accuracy	0.9716±0.0018	0.9717±0.0018	0.9702±0.0019	0.9772±0.0018	0.9695±0.0021	0.9730±0.0018
	Precision	0.9826±0.0015	0.9823±0.0013	0.9832±0.0014	0.9869±0.0012	0.9832±0.0014	0.9853±0.0013
	Recall	0.9868±0.0015	0.9872±0.0017	0.9846±0.0017	0.9884±0.0016	0.9839±0.0020	0.9855±0.0016
	F1-score	0.9847±0.0010	0.9848±0.0010	0.9839±0.0010	0.9877±0.0010	0.9836±0.0011	0.9854±0.0010
